# Supplementary material for: Conformation transitions of the polypeptide-binding pocket support an active substrate release from Hsp70s
Source: Nat Commun. 2017 Oct 31;8:1201. doi: 10.1038/s41467-017-01310-z (PMC5662698; doi:10.1038/s41467-017-01310-z)
Supplement: Supplementary file 1 — Supplementary Figures [file 41467_2017_1310_MOESM1_ESM.pdf]

Supplementary Figure 1

```
                                                                      1
hBiP        1  -------------------------MKLSLVAAMLLLLSAARAEEEDKKEDVGTVVGIDL   35
dBiP        1  ------------------------MKLCILLAVVAFVGLSLGEEKKEKDKELGTVIGIDL   36
gBiP        1  -----------------MARSWRASGSLVALAIVLSGCFFAISIAKEEAAKLGTVIGIDL   43
Kar2        1  ---MFFNRLSAGKLLVPLSVVLYALFVVILPLQNSFHSSNVLVRGADDVENYGTVIGIDL   57
hHsp70      1  -------------------------------------------------MAKAAAIGIDL   11
dHsp70      1  ----------------------------------------------------MPAIGIDL    8
gHsp70      1  ----------------------------------------------MAGKSEGSAIGIDL   14
bHsc70      1  -------------------------------------------------MSKGPAVGIDL   11
Ssa1        1  -------------------------------------------------MSKGPAVGIDL   11
hmtHsp70    1  MISASRAAAARLVGAAASRGPTAARHQDSWNGLSHEAFRLVSRRDYASEAIKGAVVGIDL   60
Ssc1        1  -----------------------MLAAKNILNRSSLSSSFRIATRLQSTKVQGSVIGIDL   37
eDnaK       1  ---------------------------------------------------MGKIIGIDL    9
dsDnaK      1  ---------------------------------------------------MGKIIGIDL    9
abDnaK      1  ---------------------------------------------------MAKIIGIDL    9
laDnaK      1  ---------------------------------------------------MSKIIGIDL    9
vhDnaK      1  ---------------------------------------------------MSKIIGIDL    9

                     2        3              4        5  A            C
hBiP       36  GTTYSCVGVFKNGRVEIIANDQGNRITPSYVAFTPEGERLIGDAAKNQLTSNPENTVFDA   95
dBiP       37  GTTYSCVGVYKNGRVEIIANDQGNRITPSYVAFTADGERLIGDAAKNQLTTNPENTVFDA   96
gBiP       44  GTTYSCVGVYKNGHVEIIANDQGNRITPSWVAFT-DSERLIGEAAKNQAAVNAERTIFDV  102
Kar2       58  GTTYSCVAVMKNGKTEILANEQGNRITPSYVAFT-DDERLIGDAAKNQVAANPQNTIFDI  116
hHsp70     12  GTTYSCVGVFQHGKVEIIANDQGNRTTPSYVAFT-DTERLIGDAAKNQVALNPQNTVFDA   70
dHsp70      9  GTTYSCVGVYQHGKVEIIANDQGNRTTPSYVAFT-DSERLIGDPAKNQVAMNPRNTVFDA   67
gHsp70     15  GTTYSCVGVWQHDRVEIIANDQGNRTTPSYVAFT-DSERLIGDAAKNQVAMNPTNTVFDA   73
bHsc70     12  GTTYSCVGVFQHGKVEIIANDQGNRTTPSYVAFT-DTERLIGDAAKNQVAMNPTNTVFDA   70
Ssa1       12  GTTYSCVGVFQHGKVEIIANDQGNRTTPSYVAFT-DTERLIGDAAKNQVAMNPTNTVFDA   70
hmtHsp70   61  GTTNSCVAVMEGKRAKVLENAEGARTTPSVVAFTADGERLVGMPAKRQAVTNPNNTFYAT  120
Ssc1       38  GTTNSAVAIMEGKVPKIIENAEGSRTTPSVVAFTKEGERLVGIPAKRQAVVNPENTLFAT   97
eDnaK      10  GTTNSCVAIMDGTTPRVLENAEGDRTTPSIIAYTQDGETLVGQPAKRQAVTNPQNTLFAI   69
dsDnaK     10  GTTNSCVSVMEGNEPVVIPNSEGKRTTPSIVAFVENGERKVGDPAKRQAITNPTKTIFSI   69
abDnaK     10  GTTNSCVAVLEGDKVKVIENAEGARTTPSIIAY-KDGEILVGQSAKRQAVTNPKNTLFAI   68
laDnaK     10  GTTNSAVAVLEGGEAKIIPNPEGARTTPSVVGF-KNGERQVGEVAKRAAITNP-NTVISI   68
vhDnaK     10  GTTNSCVSVMEGGEAVVIPNPEGNRTTPSVVAF-KNGERQVGEVAKRQAITNP-NTIQSV   68

                            D          7        8        9          E
hBiP       96  KRLIGRTWNDPSVQQDIKFLPFKVVEKKT-KPYIQVDIGGGQTKTFAPEEISAMVLTKMK  154
dBiP       97  KRLIGREWSDTNVQHDIKFFPFKVVEKNS-KPHISVDTSQG-AKVFAPEEISAMVLGKMK  154
gBiP      103  KRLIGRKFEDKEVQRDMKLVPYKIVNKDG-KPYIQVKIKDGETKVFSPEEISAMVLTKMK  161
Kar2      117  KRLIGLKYNDRSVQKDIKHLPFNVVNKDG-KPAVEVSVK-GEKKVFTPEEISGMILGKMK  174
hHsp70     71  KRLIGRKFGDPVVQSDMKHWPFQVINDGD-KPKVQVSYK-GETKAFYPEEISSMVLTKMK  128
dHsp70     68  KRLIGRKYDDPKIAEDMKHWPFKVVSDGG-KPKIGVEYK-GESKRFAPEEISSMVLTKMK  125
gHsp70     74  KRLIGRRFSDASVQSDMKLWPFTVTPGAGDKPMITVAYK-GEDKMFAAEEISSMVLMKMK  132
bHsc70     71  KRLIGRRFDDAVVQSDMKHWPFMVVNDAG-RPKVQVEYK-GETKSFYPEEVSSMVLTKMK  128
Ssa1       71  KRLIGRRFDDAVVQSDMKHWPFMVVNDAG-RPKVQVEYK-GETKSFYPEEVSSMVLTKMK  128
hmtHsp70  121  KRLIGRRYDDPEVQKDIKNVPFKIVRASN-GDAWVEAHG----KLYSPSQIGAFVLMKMK  175
Ssc1       98  KRLIGRRFEDAEVQRDIKQVPYKIVKHSN-GDAWVEARG----QTYSPAQIGGFVLNKMK  152
eDnaK      70  KRLIGRRFQDEEVQRDVSIMPFKIIAADN-GDAWVEVKG----QKMAPPQISAEVLKKMK  124
dsDnaK     70  KRFMGETFD--QVSKEVNRVPYKVVRGDN-NTPRVEIDD----RKYSPQEISAMTLQKMK  124
abDnaK     69  KRLIGRRYEDQAVQKDIGLVPYKIIKADN-GDAWVEVND----KKLAPQQISAEILKKMK  123
laDnaK     69  KRHMGTDYK-------------------------ETIEG----KDYSPQEISAIILQYLK   99
vhDnaK     69  KRHMGTDYK-------------------------VKIED----KEFTPQEVSAIILQHIK   99
```

Supplementary Figure 1

```
                     10          F         11    G
hBiP      155  ETAEAYLGKKVTHAVVTVPAYFNDAQRQATKDAGTIAGLNVMRIINEPTAAAIAYGLDKR  214
dBiP      155  ETAEAYLGKKVTHAVVTVPAYFNDAQRQATKDAGVIAGLQVMRIINEPTAAAIAYGLDKK  214
gBiP      162  ETAEAFLGKKIKDAVVTVPAYFNDAQRQATKDAGIIAGLNVARIINEPTAAAIAYGLDKK  221
Kar2      175  QIAEDYLGTKVTHAVVTVPAYFNDAQRQATKDAGTIAGLNVLRIVNEPTAAAIAYGLDKS  234
hHsp70    129  EIAEAYLGYPVTNAVITVPAYFNDSQRQATKDAGVIAGLNVLRIINEPTAAAIAYGLDRT  188
dHsp70    126  ETAEAYLGESITDAVITVPAYFNDSQRQATKDAGHIAGLNVLRIINEPTAAALAYGLDKN  185
gHsp70    133  ETAEAYLGSSVKNGVVTVPAYFNDSQRQATKDAGVIAGLNVMRIINEPTAAAIAYGLDKK  192
bHsc70    129  EIAEAYLGKTVTNAVVTVPAYFNDSQRQATKDAGTIAGLNVLRIINEPTAATIAYGLDKK  188
Ssa1      129  EIAEAYLGKTVTNAVVTVPAYFNDSQRQATKDAGTIAGLNVLRIINEPTAATIAYGLDKK  188
hmtHsp70  176  ETAENYLGRTAKNAVITVPAYFNDSQRQATKDAGQISGLNVLRVINEPTAAALAYGLDKS  235
Ssc1      153  ETAEAYLGKPVKNAVVTVPAYFNDSQRQATKDAGQIVGLNVLRVVNEPTAAALAYGLEKS  212
eDnaK     125  KTAEDYLGEPVTEAVITVPAYFNDAQRQATKDAGRIAGLEVKRIINEPTAAALAYGLDKG  184
dsDnaK    125  KTAEDYLGQEVTEAVVTVPAYFSDSQRQATKEAGEIAGLKVRRIINEPTAASLAYGLDKM  184
abDnaK    124  KTAEDYLGETVTEAVITVPAYFNDAQRQATKDAGKIAGLDVKRIINEPTAAALAFGMDKK  183
laDnaK    100  GYAEDYLGETVDKAVITVPAYFNDAQRQATKDAGKIAGLEVERIINEPTAAALAYGMDKT  159
vhDnaK    100  SYAEDYIGETVEKAVITVPAYFNDAERQATKDAGKIAGLEVERIINEPTAAALAYGIDKD  159

                     12       13            14            H
hBiP      215  E---GEKNILVFDLGGGTFDVSLLTI---DNG-VFEVVATNGDTHLGGEDFDQRVMEHFI  267
dBiP      215  E---GEKNVLVFDLGGGTFDVSLLTI---DNG-VFEVVATNGDTHLGGEDFDQRVMDHFI  267
gBiP      222  G---GEKNILVFDLGGGTFDVSILTI---DNG-VFEVLSTNGDTHLGGEDFDQRIMEYFI  274
Kar2      235  D---KEHQIIVYDLGGGTFDVSLLSI---ENG-VFEVQATSGDTHLGGEDFDYKIVRQLI  287
hHsp70    189  GK--GERNVLIFDLGGGTFDVSILTI---DDG-IFEVKATAGDTHLGGEDFDNRLVNHFV  242
dHsp70    186  LK--GERNVLIFDLGGGTFDVSILTI---DEGSLFEVRSTAGDTHLGGEDFDNRLVTHLA  240
gHsp70    193  ASSVGEKNVLIFDLGGGTFDVSLLTI---EEG-IFEVKATAGDTHLGGEDFDNRMVNHFV  248
bHsc70    189  VG--AERNVLIFDLGGGTFDVSILTI---EDG-IFEVKSTAGDTHLGGEDFDNRMVNHFI  242
Ssa1      189  VG--AERNVLIFDLGGGTFDVSILTI---EDG-IFEVKSTAGDTHLGGEDFDNRMVNHFI  242
hmtHsp70  236  E----DKVIAVYDLGGGTFDISILEI---QKG-VFEVKSTNGDTFLGGEDFDQALLRHIV  287
Ssc1      213  D----SKVVAVFDLGGGTFDISILDI---DNG-VFEVKSTNGDTHLGGEDFDIYLLREIV  264
eDnaK     185  T---GNRTIAVYDLGGGTFDISIIEIDEVDGEKTFEVLATNGDTHLGGEDFDSRLINYLV  241
dsDnaK    185  D---RDMKIAVFDLGGGTFDISILEL---GDG-VFEVKSTDGDTHLGGDDFDQVIIDWLA  237
abDnaK    184  E---GDRKVAVYDLGGGTFDVSIIEIADLDGDQQIEVLSTNGDTFLGGEDFDNALIEYLV  240
laDnaK    160  D---KDQTILVFDLGGGTFDVSILEL---GDG-VFEVHSTAGDNHLGGDDFDQKIIDYLV  212
vhDnaK    160  D---QDQTILVYDLGGGTFDVSILDI---GDG-TFEVVSTAGDNRLGGDDFDEVIINHMV  212

                                I          15          16
hBiP      268  KLYKKKTGKDVRKDNRAVQKLRREVEKAKRALSSQHQARIEIESFYE----GEDFSETLT  323
dBiP      268  KLYKKKKGKDIRKDNRAVQKLRREVEKAKRALSGSHQVRIEIESFFE----GDDFSETLT  323
gBiP      275  KLIKKKHGKDISKDNRALGKLRREAERAKRALSSQHQVRVEIESLFD----GVDFSEPLT  330
Kar2      288  KAFKKKHGIDVSDNNKALAKLKREAEKAKRALSSQMSTRIEIDSFVD----GIDLSETLT  343
hHsp70    243  EEFKRKHKKDISQNKRAVRRLRTACERAKRTLSSSTQASLEIDSLFE----GIDFYTSIT  298
dHsp70    241  DEFKRKYKKDLRSNPRALRRLRTAAERAKRTLSSSTEATIEIDALFE----GQDFYTKVS  296
gHsp70    249  QEFKRKNKKDISGNPRALRRLRTSCERAKRTLSSTAQTTIEIDSLYE----GIDFYSTIT  304
bHsc70    243  AEFKRKHKKDISENKRAVRRLRTACERAKRTLSSSTQASIEIDSLYE----GIDFYTSIT  298
Ssa1      243  AEFKRKHKKDISENKRAVRRLRTACERAKRTLSSSTQASIEIDSLYE----GIDFYTSIT  298
hmtHsp70  288  KEFKRETGVDLTKDNMALQRVREAAEKAKCELSSSVQTDINLPYLTMDSSGPKHLNMKLT  347
Ssc1      265  SRFKTETGIDLENDRMAIQRIREAAEKAKIELSSTVSTEINLPFITADASGPKHINMKFS  324
eDnaK     242  EEFKKDQGIDLRNDPLAMQRLKEAAEKAKIELSSAQQTDVNLPYITADATGPKHMNIKVT  301
dsDnaK    238  DEFNSEEGIDLRKDPMALQRLKEAAEKAKIELSSSSQTEINLPYIMPVNGIPKHLVKTLS  297
abDnaK    241  EEFKKEQNVNLKNDPLALQRLKEAAEKAKIELSSSNATEINLPYITADATGPKHLVINVT  300
laDnaK    213  SEFKKENGIDLSQDKMALQRLKDAAEKAKKDLSGVMSTQISLPFITAGEAGPLHLEVNLT  272
vhDnaK    213  QEFKKENGIDLSKDKMATQRLKDAAEKAKKDLSGVSQTQISLPFITAGEAGPLHLEMNLT  272
```

Supplementary Figure 1

```
                J/K                 L   17        N
hBiP      324  RAKFEELNMDLFRSTMKPVQKVLEDSDLKKSDIDEIVLVGGSTRIPKIQQLVKEFFNGKE  383
dBiP      324  RAKFEELNLDLFRSTLKPVQKVLEDADMNKKDVHEIVLVGGSTRIPKVQQLVKDFFGGKE  383
gBiP      331  RARFEELNNDLFRKTMGPVKKAMEDAGLQKSQIDEIVLVGGSTRIPKVQQLLKDYFDGKE  390
Kar2      344  RAKFEELNLDLFKKTLKPVEKVLQDSGLEKKDVDDIVLVGGSTRIPKVQQLLESYFDGKK  403
hHsp70    299  RARFEELCSDLFRSTLEPVEKALRDAKLDKAQIHDLVLVGGSTRIPKVQKLLQDFFNGRD  358
dHsp70    297  RARFEELCADLFRNTLQPVEKALNDAKMDKGQIHDIVLVGGSTRIPKVQSLLQDFFHGKN  356
gHsp70    305  RARFEELNMDLFRKCMEPVEKCLRDAKMDKSTVHDVVLVGGSTRIPKVQQLLQDFFNGKE  364
bHsc70    299  RARFEELNADLFRGTLDPVEKALRDAKLDKSQIHDIVLVGGSTRIPKIQKLLQDFFNGKE  358
Ssa1      299  RARFEELNADLFRGTLDPVEKALRDAKLDKSQIHDIVLVGGSTRIPKIQKLLQDFFNGKE  358
hmtHsp70  348  RAQFEGIVTDLIRRTIAPCQKAMQDAEVSKSDIGEVILVGGMTRMPKVQQTVQDLF-GRA  406
Ssc1      225  RAQFETLTAPLVKRTVDPVKKALKDAGLSTSDISEVLLVGGMSRMPKVVETVKSLF-GKD  383
eDnaK     302  RAKLESLVEDLVNRSIEPLKVALQDAGLSVSDIDDVILVGGQTRMPMVQKKVAEFF-GKE  360
dsDnaK    298  RAKFEQLADSLINATIEPCRKALKNAGMTASDIDEVILVGGSTRIPAIQDKVKEFF-GKE  356
abDnaK    301  RAKLEGLVGDLVARTIEPCKIALKDAGLSTSDISDVILVGGQSRMPLVQQKVQEFF-GRE  359
laDnaK    273  RAKFDELTSDLVERTVGPTRQALKDAGLSASDIDQVILVGGSTRIPAVQEAITKEL-GKE  331
vhDnaK    273  RAKFEELSSDLVERTMVPTRKALSDASLSANDIHKVILVGGSTRIPAVQEAIKREI-GQE  331

                      O                 βL     β1     β2
hBiP      384  PSRGINPDEAVAYGAAVQAGVLSGD--QDTGDLVLLHVCPLTLGIETVGGVMTKLIPSNT  441
dBiP      384  PSRGINPDEAVAYGAAVQAGVLSGE--QDTDAIVLLDVNPLTMGIETVGGVMTKLIPRNT  441
gBiP      391  PNKGVNPDEAVAYGAAVQGGILSGEGGDETKDILLLDVAPLTLGIETVGGVMTKLIPRNT  450
Kar2      404  ASKGINPDEAVAYGAAVQAGVLSGE--EGVEDIVLLDVNALTLGIETTGGVMTPLIKRNT  461
hHsp70    358  LNKSINPDEAVAYGAAVQAAILMGDKSENVQDLLLLDVAPLSLGLETAGGVMTALIKRNS  418
dHsp70    357  LNLSINPDEAVAYGAAVQAAILSGDQSGKIQDVLLVDVAPLSLGIETAGGVMTKLIERNC  416
gHsp70    365  LCKSINPDEAVAYGAAVQAAILSGEGNEKVQDLLLLDVTPLSLGLETAGGVMTVLIPRNT  424
bHsc70    359  LNKSINPDEAVAYGAAVQAAILSGDKSENVQDLLLLDVTPLSLGIETAGGVMTVLIKRNT  418
Ssa1      359  LNKSINPDEAVAYGAAVQAAILSGDKSENVQDLLLLDVTPLSLGIETAGGVMTVLIKRNT  418
hmtHsp70  407  PSKAVNPDEAVAIGAAIQGGVLAG----DVTDVLLLDVTPLSLGIETLGGVFTKLINRNT  462
Ssc1      384  PSKAVNPDEAVAIGAAVQGAVLSG----EVTDVLLLDVTPLSLGIETLGGVFTRLIPRNT  439
eDnaK     361  PRKDVNPDEAVAIGAAVQGGVLTG----DVKDVLLLDVTPLSLGIETMGGVMTTLIAKNT  416
dsDnaK    357  ASKGVNPDEVVAIGAAIQGGVLTG----EVKDVLLLDVTPLSLGIETMGGVMTKLIESNT  412
abDnaK    360  PRKDVNPDEAVAIGAAIQGAVLSG----DKNDVLLLDVTPLTLGIETMGGVLTPIIEKNT  415
laDnaK    332  PHRGVNPDEVVAMGAAIQGGVITG----DVKDVVLLDVTPLSLGIETMGGVMTSLIERNT  387
vhDnaK    332  PSKGVNPDEVVALGAAIQGGVLQG----DVKDVVLLDVTPLSLGIETMGSVFTKLIERNT  387

                   β3        β4            β5               β6
hBiP      442  VVPTKNSQIFSTASDNQPTVTIKVYEGERPLTKDNHLLGTFDLTGIPPAPRGVPQIEVTF  501
dBiP      442  VIPTKKSQVFSTASDNQHTVTIQVYEGERPMTKDNHLLGKFDLTGIPPAPRGIPQIEVSF  501
gBiP      451  VIPTKKSQVFTTYQDQQTTVSIQVFEGERSLTKDCRLLGKFDLTGIPPAPRGTPQIEVTF  510
Kar2      462  AIPTKKSQIFSTAVDNQPTVMIKVYEGERAMSKDNNLLGKFELTGIPPAPRGVPQIEVTF  521
hHsp70    419  TIPTKQTQIFTTYSDNQPGVLIQVYEGERAMTKDNNLLGRFELSGIPPAPRGVPQIEVTF  478
dHsp70    417  RIPCKQTKTFSTYADNQPGVSIQVYEGERAMTKDNNALGTFDLSGIPPAPRGVPQIEVTF  476
gHsp70    425  TIPTKKEQVFSTYSDNQPGVLIQVYEGERTRTRDNNLLGKFELSGIPPAPRGVPQITVCF  484
bHsc70    419  TIPTKQTQTFTTYSDNQPGVLIQVYEGERAMTKDNNLLGKFELTGIPPAPRGVPQIEVTF  478
Ssa1      419  TIPTKQTQTFTTYSDNQPGVLIQVYEGERAMTKDNNLLGKFELTGIPPAPRGVPQIEVTF  478
hmtHsp70  463  TIPTKKSQVFSTAADGQTQVEIKVCQGEREMAGDNKLLGQFTLIGIPPAPRGVPQIEVTF  522
Ssc1      440  TIPTKKSQIFSTAAAGQTSVEIRVFQGERELVRDNKLIGNFTLAGIPPAPKGVPQIEVTF  499
eDnaK     417  TIPTKHSQVFSTAEDNQSAVTIHVLQGERKRAADNKSLGQFNLDGINPAPRGMPQIEVTF  476
dsDnaK    413  TIPTKKSETFTTAADNQPSVEIHVLQGERPIASGNKTIGRFHLDGIPPSPRGIPQIEVTF  472
abDnaK    416  TIPAKKSQVFSTAADNQPAVDISVYQGERKMAQQNKLLGNFQLGDIPPAPRGVPQIEVSF  475
laDnaK    388  TIPTSKSQVFSTAADNQPAVDIHVLQGERPMAKDNKTLGRFQLADIPAAPRGVPQIEVSF  447
vhDnaK    388  TIPTSHSQVFSTAADNQTAVDIHVLQGEREMAADNKTLGRFQLTDIPPAPRGVPQIEVSF  447
```

Supplementary Figure 1

β7 β8 αA/B

hBiP	502	EIDVNGILRVTAEDKGTGNKNKITITNDQNRLTPEEIERMVNDAEKFAEEDKKLKERIDT	561
dBiP	502	EIDANGILQVSAEDKGTGNKEKIVITNDQNRLTPEDIDRMIRDAEKFADEDKKLKERVES	561
gBiP	511	EVDANGILNVKAEDKGTGKSEKITITNDKGRLSQEEIERMVREAEEFAEEDKKVKERIDA	570
Kar2	522	ALDANGILKVSATDKGTGKSESITITNDKGRLTQEEIDRMVEEAEKFASEDASIKAKVES	581
hHsp70	479	DIDANGILNVTATDKSTGKANKITITNDKGRLSKEEIERMVQEAEKYKAEDEVQRERVSA	538
dHsp70	477	DLDANGILNVSAKEMSTGKAKNITIKNDKGRLSQAEIDRMVNEAEKYADEDEKHRQRITS	536
gHsp70	485	DIDANGILNVSAEDKTTGQKNKITITNDKGRLSKEEIEKMVQEAEKYKSEDEEHKKKVEA	544
bHsc70	479	DIDANGILNVSAVDKSTGKENKITITNDKGRLSKEDIERMVQEAEKYKAEDEKQRDKVSS	538
Ssa1	479	DIDANGILNVSAVDKSTGKENKITITNDKGRLSKEDIERMVQEAEKYKAEDEKQRDKVSS	538
hmtHsp70	523	DIDANGIVHVSAKDKGTGREQQIVIQSSGG-LSKDDIENMVKNAEKYAEEDRRKKERVEA	581
Ssc1	500	DIDADGIINVSARDKATNKDSSITVAGSSG-LSENEIEQMVNDAEKFKSQDEARKQAIET	558
eDnaK	477	DIDADGILHVSAKDKNSGKEQKITIKASSG-LNEDEIQKMVRDAEANAEADRKFEELVQT	535
dsDnaK	473	DIDANGILHVHAKDKATGKSQSIRIEASSG-LSDDEINRMKSEAEANAEADKKAKETADK	531
abDnaK	476	DINADGILKVSAKDKSTGKEQSIQIKANSG-LSDAEIEAMIKDAEANAEEDRKFEELAKA	534
laDnaK	448	DIDKNGIVTVRAKDLGTGKEQNIVIKSSSG-LTDDEIDRMVKDAEANAEEDKKNKENVET	506
vhDnaK	448	DIDANGIVNVRAKDMGTNKEQSITIKSSSG-LSDEEVDRMVKEAEENAEEDKKRREEIEL	506

αC αD/E

hBiP	562	RNELESYAYSLKNQIGDKEKLGGKLSSEDKETMEKAVEEKIEWLES---HQDADIEDFKA	618
dBiP	562	RNELESYAYSLKNQIGDKDKLGAKLSDDEKNKLESAIDESIKWLEQ---NPDADPEEYKK	618
gBiP	571	RNSLETYIYNMKNQINDKDKLADKLESDEKEKVETAVKEALEWLDD---NQSAEKEDYEE	627
Kar2	582	RNKLENYAHSLKNQVNGDLGE--KLEEEDKETLLDAANDVLEWLDD--NFETAIAEDFDE	637
hHsp70	539	KNALESYAFNMKSAVEDEGLKG-KISEADKKKVLDKCQEVISWLDA---NTLAEKDEFEH	594
dHsp70	537	RNALESYVFNVKQAVEQAPAG--KLDEADKNSVLDKCNDTIRWLDS---NTTAEKEEFDH	591
gHsp70	545	KNALENYAYNMRNTVKDEKIGA-KLNPADKKRIEDAVEQTINWLDS---NQLAEADEFED	600
bHsc70	539	KNSLESYAFNMKATVEDEKLQG-KINDEDKQKILDKCNEIINWLDK---NQTAEKEEFEH	594
Ssa1	539	KNSLESYAFNMKATVEDEKLQG-KINDEDKQKILDKCNEIINWLDK---NQTAEKEEFEH	594
hmtHsp70	582	VNMAEGIIHDTETKMEEFKD---QLPADECNKLKEEISKMRELLAR---KDSETGENIRQ	635
Ssc1	559	ANKADQLANDTENSLKEFEG---KVDKAEAQKVRDQITSLKELVARVQGGEEVNAEELKT	615
eDnaK	536	RNQGDHLLHSTRKQVEEAGD---KLPADDKTAIESALTALETALKG------EDKAAIEA	586
dsDnaK	532	INQADSLIFQTEKQLKEYGD---KLPADKKGPIEDALKKLKEAHAS------KDLAAIEA	582
abDnaK	535	RNEADALISSSNKAVKDLGD---KVTEDEKTAVNTAVSELEAATKE------NDVEAIKA	585
laDnaK	507	RNNADQLIFTVDKTLKELEG---KVDADEVKKAETARDELQEALKG------EDFDAIKE	557
vhDnaK	507	RNEADQLIFTTDKTIKDLGE---KVSDEEKEKAEKAKEELKSALES------DDQEQIKE	557

αD/E

hBiP	619	KKKELEEIVQPIISKLYGSAGPPPTGEEDTAE---------KDEL	654
dBiP	619	QKKDLEAIVQPVIAKLYQGAGGAPPPEGGDDADL-------KDEL	656
gBiP	628	KLKEVEAVCNPIITAVYQRSGGAPGGGSTEEDDDS------HDEL	666
Kar2	638	KFESLSKVAYPITSKLYGGADGSGAADYDDEDEDDDGDYFEHDEL	682
hHsp70	595	KRKELEQVCNPIISGLYQGAGGP-GPGGFGAQGPKGGSGSGPTIEEVD	641
dHsp70	592	KLEELTRHCSPIMTKMHQQGAGAGAGGPGANCGQQAGGFGGYSGPTVEEVD	642
gHsp70	601	KMKELENMCNPIIAKMYQGAGGEPAAGKAGEAPPAGGSGAGPKIEEVD	648
bHsc70	595	QQKELEKVCNPIITKLYQSAGGMPGGMPGGMPGGFPGGGAPPSGGASSGPTIEEVD	650
Ssa1	595	QQKELEKVCNPIITKLYQSAGGMPGGMPGGMPGGFPGGGAPPSGGASSGPTIEEVD	650
hmtHsp70	636	AASSLQQASLKLFEMAYKKMASEREGSGSSGTGEQKEDQKEEKQ	679
Ssc1	616	KTEELQTSSMKLFEQLYKNDSNNNNNNNGNNAESGETKQ	654
eDnaK	587	KMQELAQVSQKLMEIAQQQHAQQQTAGADASANNAKDDDVVDAEFEEVKDKK	638
dsDnaK	583	AMNELNTVFQAASQEMYNASQAQGGAQPGPDAGQQAGPQDQPKDDGEVTDVDFEEVK	637
abDnaK	586	KTEALQNILMPITQRAYEQAQQAGGAEGFDPNAFQGGDAGQQKADDGVVDAEFTEVKDDK	646
laDnaK	558	KSDALNEIVQSLSVKLYEQAAAAQQAQGENPEANAADGSEDVVDADFEEINDDDKKEDK	615
vhDnaK	558	KKEALEEQVQQLSVKMYEQMQQEAQAQQGQEDQGSDDDVVDADYQEVDEEKDEKDNK	613

Supplementary Figure 1

Supplementary Figure 1. Structure-based sequence alignment of representative Hsp70s. The amino acid sequence of human BiP (hBiP) is aligned with the sequences of a number of selected Hsp70s from both eukaryotes (different cellular compartments) and prokaryotes. The BiP construct used in crystallization is from residue 25 to 633, and all the residues in the construct (highlighted in red) are ordered in both protomers. $L_{3,4}$, which is mutated to VGG ($L_{3,4}'$), is highlighted in blue. Elements of secondary structure in the BiP-ATP2 structure are specified by cylinders for helices and arrows for strands and labeled. The coloring of structural elements is: NBD (blue), Linker (purple), SBDβ (green) and SBDα (red). The highly conserved Gly425, Gly430 and Gly431 are highlighted in purple. The first four sequences are Hsp70s from endoplasmic reticulum (ER); the next 5 sequences (names in red) are Hsp70s from eukaryotic cytosol; the following two sequences (names in green) are Hsp70s from mitochondria; and the last five sequences are Hsp70s from prokaryotes (names in blue). hBiP: human ER Hsp70; dBiP: Drosophila melanogaster ER Hsp70; gBiP: Gossypium hirsutum ER Hsp70; Kar2: *S. cerevisiae* ER Hsp70; hHsp70: human Hsp70; dHsp70: Drosophila melanogaster Hsp70; gHsp70: Gossypium hirsutum Hsp70; bHsc70: bovine Hsc70; Ssa1: *S. cerevisiae* Hsp70; hmtHsp70: human mitochondrial Hsp70; bmtHsp70: bovine mitochondrial Hsp70; Ssc1: *S. cerevisiae* mitochondrial Hsp70; eDnaK: *Escherichia coli* Hsp70 (gram-negative); dsDnaK: Draconibacterium sp. Hsp70 (gram-negative); abDnaK: Acinetobacter baumannii Hsp70 (gram-negative); laDnaK: Listeria aquatica Hsp70 (gram-positive); vhDnaK: Virgibacillus halodenitrificans Hsp70 (gram-positive).

Supplementary Figure 2

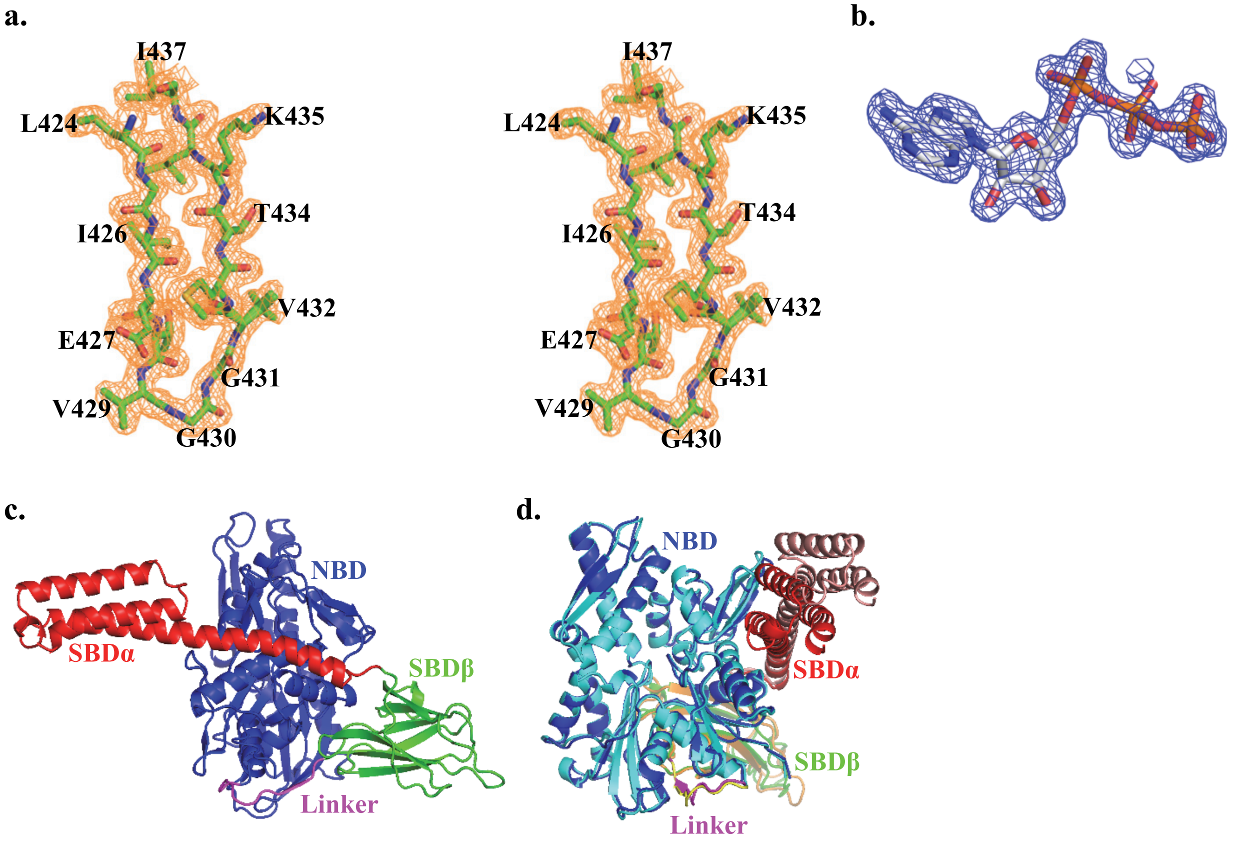

e.

rmsd (Å)	BiP-ATP2/BiP-ATP	BiP-ATP2/DnaK-ATP
NBD	0.513	0.920
SBDβ	2.337	2.618
SBDβ-core	0.591	0.615
SBDα	1.076	2.174

SBDβ-core: SBDβ excluding $L_{1,2}$, $L_{3,4}$, $L_{4,5}$, $L_{5,6}$, and β8.

f.

	k_{cat} (x10^{-3} min^{-1})
- Pi	12.92±0.92
+ Pi	2.55±0.36

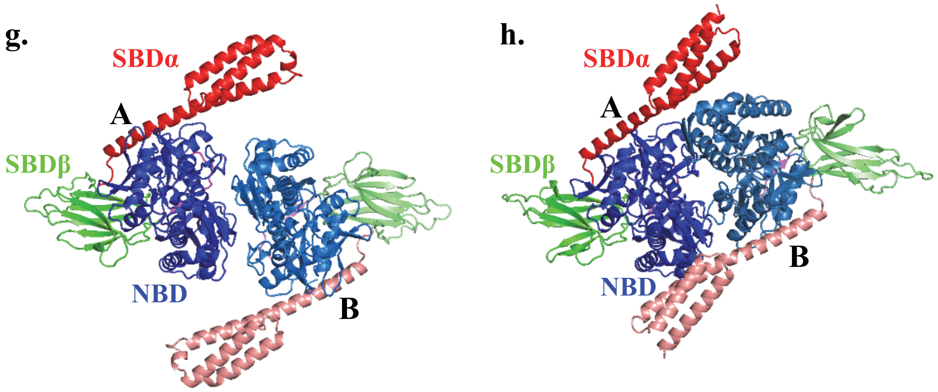

Supplementary Figure 2

Supplementary Figure 2. The BiP-ATP2 structure and structural comparison.
a-b, Electron-density maps for $L_{1,2}$ (**a**, stereo image) and ATP (**b**) of the BiP-ATP2 structure. 2Fo-Fc maps (contoured at 1σ) were shown as meshes. $L_{1,2}$ and ATP were drawn as sticks.
c, The BiP-ATP2 structure. Orthogonal view of Fig. 1**b**.
d, Structural comparison of BiP-ATP2 with BiP-ATP (PDB code: 5E84). Orthogonal view of Fig. 1**c**.
e, Cα root-mean-square deviation (rmsd) between the Hsp70-ATP structures. NBD and SBDβ-core have the smallest rmsd.
f, Phosphate reduces the ATPase activity of BiP. Single-turnover ATPase assay was carried with BiP protein in the presence of 100 mM phosphate (+ Pi), and the catalytic constant (k_{cat}) was calculated after fitting the data using Prism. The ATPase activity in the absence of phosphate (- Pi) was used as a control.
g-h, Ribbon diagrams of BiP-ATP2 dimer (**g**) and DnaK-ATP-dimer (**h**, PDB code: 4JNE). Protomer As from BiP-ATP2 and DnaK-ATP are superimposed. The domain coloring for protomer A is the same as Fig. 1**b**, and the domain coloring for protomer B is: NBD (light blue), SBDβ (light green), SBDα (pink), and inter-domain linker (magenta).

Supplementary Figure 3

a.

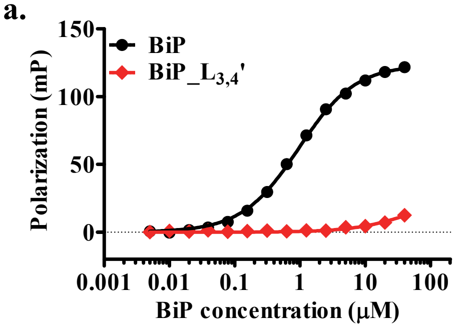

b. **c.** **d.**

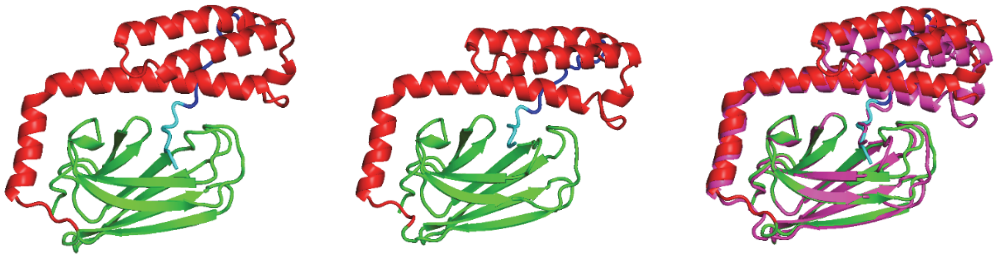

BiP-SBD **BiP-SBD-$L_{3,4}$'** **Superposition rmsd for SBDβ=0.723 Å**

Supplementary Figure 3. The $L_{3,4}$' modification in BiP significantly compromises peptide substrate binding but has little structural impact in the isolated domain structure.

a, Peptide substrate binding to BiP proteins using fluorescence polarization. The NR peptide was labeled with fluorescein at the N-terminus. This F-NR peptide was incubated with serial dilutions of BiP proteins. After binding reached equilibrium, fluorescence polarization was measured and fitted to one-site binding equation. The dissociation constant (K_d) for the WT BiP is 0.95 μM, consistent with published results. The binding for BiP-$L_{3,4}$' protein was drastically reduced.

b-d, Structural comparison of the isolated SBD domain of BiP. The structures of isolated SBD from WT BiP (**b**, PDB code: 5E85) and BiP-$L_{3,4}$' (**c**, PDB code: 5E86) were superimposed based on the Cαs of SBDβ. The coloring for both structures is: SBDβ (green), SBDα (red), linked NR (cyan), and the Tev linker between SBD and NR peptide (blue). **d** is the superposition of the two structures. The WT SBD structure has the same color code as in **b** and the SBD-$L_{3,4}$' is colored in purple. The rmsd for the SBDβ is 0.723Å.

Supplementary Figure 4

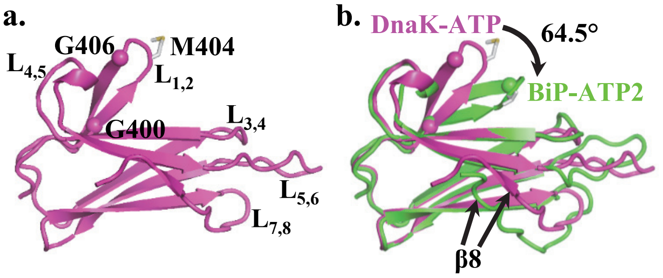

c. Cα distance (Å)

	BiP-ATP2 vs BiP-ATP	BiP-ATP vs DnaK-ATP
T428	5.01	4.98
V429	6.93	6.66
G430	9.24	9.73
G431	10.30	10.83
V432	7.58	8.87
M433	5.09	5.80

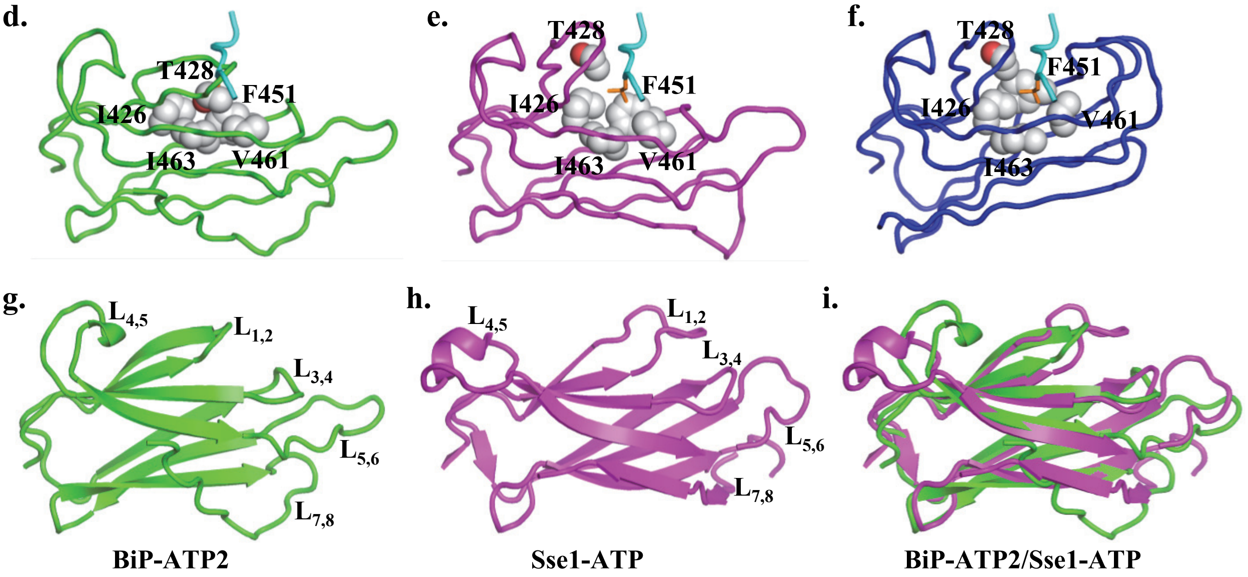

Supplementary Figure 4. The conformations of the polypeptide-binding pocket.

a, Ribbon diagram of the SBDβ from the DnaK-ATP structure (PDB code: 4JNE). The Cα atoms of Gly400 and Gly406 are shown as balls.

b, Superposition of the SBDβs from BiP-ATP2 (green) and DnaK-ATP (purple). The structures were superimposed based on the Cα atoms of the SBDβ-core. The Cα atoms of Gly400 and Gly406 are shown as balls. The rotation of $L_{1,2}$ between BiP-ATP2 and DnaK-ATP is labeled.

c, Distances between the Cα atoms in the β1-$L_{1,2}$-β2 segment from the BiP-ATP2, BiP-ATP and DnaK-ATP structures.

d-f, The polypeptide-binding pockets in BiP-ATP2 (**d**), BiP-ATP (**e**, PDB code: 5E84), and isolated BiP SBD (**f**, PDB code: 5E85). SBDβs are shown as worm representation. The NR peptide bound to the isolated SBD is shown in cyan with the side chain of the central leucine, Leu4, highlighted in yellow. SBDβ side chains that make van der Waals contacts with Leu4 in the isolated BiP SBD structure are shown as sphere drawings. Since the polypeptide-binding pocket is fully closed, these residues pack against each other to exclude the binding of the NR peptide.

g-i, Structural comparison of the SBDβs from BiP-ATP2 (**g**) and Sse1-ATP (**h**, PDB code: 2QXL). **i** is the superposition of **g** and **h**. The SBDβs are superimposed based on Cα atoms. Except $L_{4,5}$ and β8, the two structures aligned well (rmsd: 1.511 Å), especially the peptide-binding loops. The disordered region in $L_{7,8}$ of Sse1-ATP is not shown.

Supplementary Figure 5

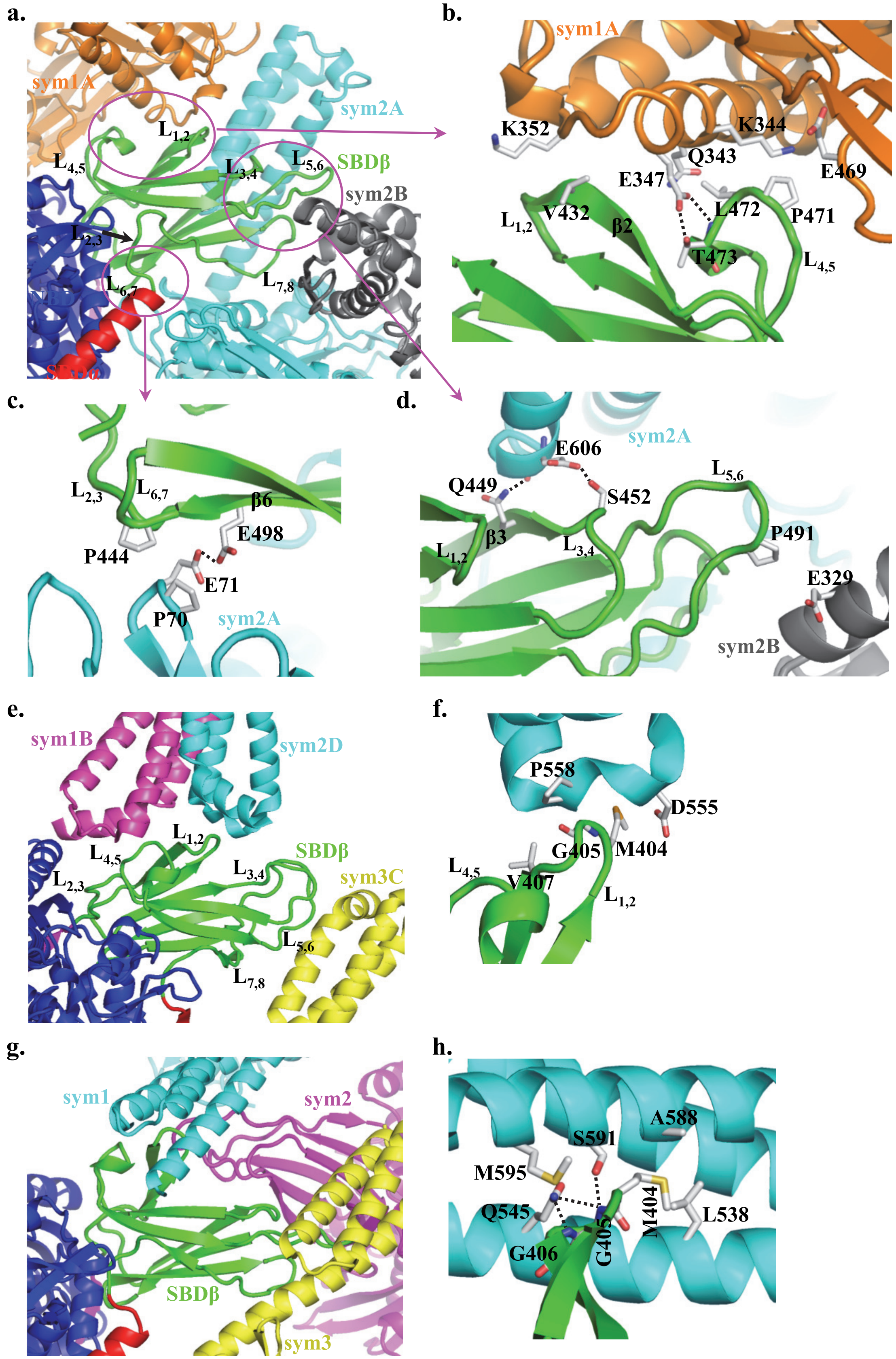

Supplementary Figure 5

Supplementary Figure 5. Crystal contacts surrounding the SBDβ region.
a-d, Crystal contacts in the BiP-ATP2 structure. **a**, Overall crystal contacts surrounding the SBDβ. Only protomer A was shown (NBD: blue; SBDβ: green; and SBDα: red). Three molecules from two symmetry mates form crystal contacts with SBDβ: protomer A from symmetry mate 1 (sym1A, orange), and both protomers from symmetry mate 2 (sym2A and sym2B, cyan and grey, respectively). **b**, Details of the crystal contacts formed between SBDβ and sym1A. $L_{1,2}$ and $L_{4,5}$ of SBDβ are involved in these crystal contacts. A hydrophobic contact was formed between Val432 (in $L_{1,2}$) and Lys352 (sym1A). Two hydrogen bonds were formed between the side-chain of Glu347 (sym1A) and Thr473 in $L_{4,5}$ (both side-chain and main-chain). There are a number of hydrophobic contacts formed between two residues on $L_{4,5}$ (Lys472 and Pro471) and three residues on sym1A (Gln343, Lys344 and Glu469). **c**, Details of the crystal contacts formed between SBDβ (the $L_{2,3}$ and $L_{6,7}$ region) and sym2A. Hydrophobic contacts were formed between the side-chains of Pro444 (in $L_{2,3}$) and Pro70 and Glu71 from sym2A. Moreover, a hydrogen bond was formed between Glu498 (in β6) and Glu71 in sym2A. **d**, Details of the crystal contacts formed between SBDβ and sym2A and sym2B around the $L_{3,4}$ and $L_{5,6}$ region. There are two hydrogen bonds: between Ser452 in $L_{3,4}$ and Glu606 of Sym2A, and between Gln449 in β3 and Glu606 of Sym2A. Hydrophobic contacts were formed between Pro491 in $L_{5,6}$ and side-chain of Glu329 from sym2B.
e-f, Crystal contacts around the peptide-binding loops in the DnaK-ATP structure solved by the Mayer's group (PDB code: 4B9Q). Only protomer A was shown (NBD: blue; SBDβ: green; and SBDα: red). **e**, Overall crystal contacts around the peptide-binding loops. Three symmetry mates form crystal contacts around the peptide-binding loops: sym1B (purple), sym2D (cyan), and sym3C (yellow). **f**, $L_{1,2}$ forms crystal contacts with symmetry mate sym2D. This crystal contact features a number of hydrophobic interactions between the side-chain of Met404 in $L_{1,2}$ and side-chains of both Asp555 and Pro558 (sym2D), between the main-chain of Gly405 in $L_{1,2}$ and side-chain of Pro558 (sym2D), and between the side-chains of Val407 in $L_{1,2}$-β2 junction and Pro558 (sym2D).
g-h, Crystal contacts around the peptide-binding loops in the DnaK-ATP structure solved by our group (PDB code: 4JNE). Only protomer A was shown (NBD: blue; SBDβ: green; and SBDα: red). **g**, Overall crystal contacts around the peptide-binding loops. Three symmetry mates form crystal contacts around the peptide-binding loops: sym1 (purple), sym2 (cyan), and sym3 (yellow). **h**, The crystal contacts formed between $L_{1,2}$ and symmetry mate sym1. A number of hydrophobic interactions and hydrogen bonds are formed on these crystal contacts. The hydrogen bonds are labeled as dotted lines. The side-chain of Met404 in $L_{1,2}$ forms hydrophobic contacts with the side-chains of Leu538, Ala588 and Ser591 on sym1. The main-chain Cα of Gly406 in $L_{1,2}$ forms hydrophobic contact with Met595 on sym1.

Supplementary Figure 6

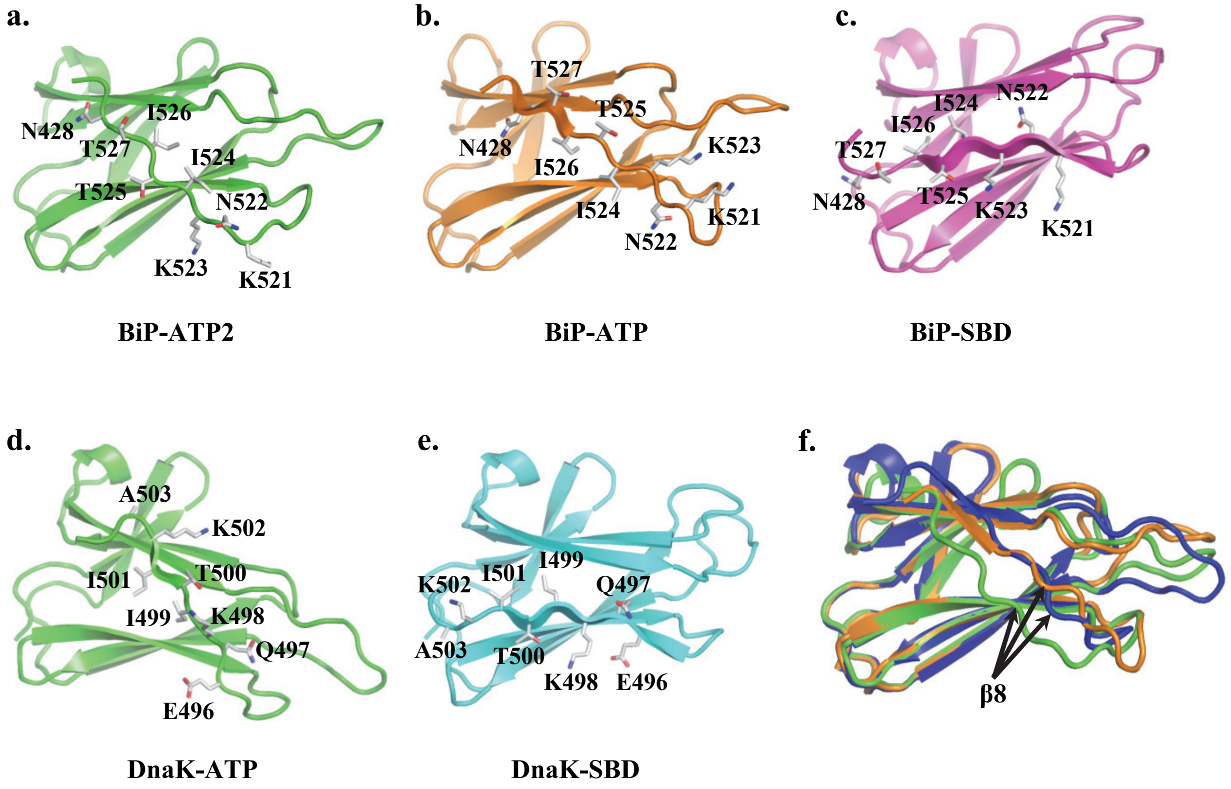

Supplementary Figure 6. The conformation of β8.

a-e, The SBDβs from BiP-ATP2 (**a**), BiP-ATP (**b**, PDB code: 5E84), BiP-SBD (**c**, PDB code: 5E85), DnaK-ATP (**d**, PDB code: 4JNE), and DnaK-SBD (**e**, PDB code: 1DKX) are drawn in ribbon diagram. Side chains of β8 are highlighted in stick representation. The β8 in BiP-ATP2 has similar side-chain orientations as those in the isolated BiP-SBD; whereas the side-chain orientations of β8 in BiP-ATP are flipped. The side-chain orientations of β8 in the isolated DnaK SBD are similar to those of the isolated BiP SBD. Interestingly, the first two residues at the N-terminal end of β8 in DnaK-ATP have similar side-chain orientations as the isolated SBD structures, whereas for the rest of β8 starting from the third residue, the side-chain orientations are flipped as those in BiP-ATP. Thus, the side-chain orientations of β8 in DnaK-ATP seem to be a hybrid between BiP-ATP and BiP-ATP2.

f, Superposition of SBDβs from BiP-ATP2 (green), BiP-ATP (orange) and DnaK-ATP (blue). The SBDβs were superimposed based on the Cα atoms of SBDβ-core. β8 is indicated by arrows.

Supplementary Figure 7

a.

BiP proteins	k_{cat} (x10^{-3} min^{-1})	K_d (μM)
WT	12.04±0.80	0.91±0.042
C41A/C420A	12.46±0.43	0.85±0.012
C41A/C420A/V429C	10.47±0.74	0.82±0.026
C41A/C420A/V429C/T462C	9.11±1.38	0.61±0.023

b.

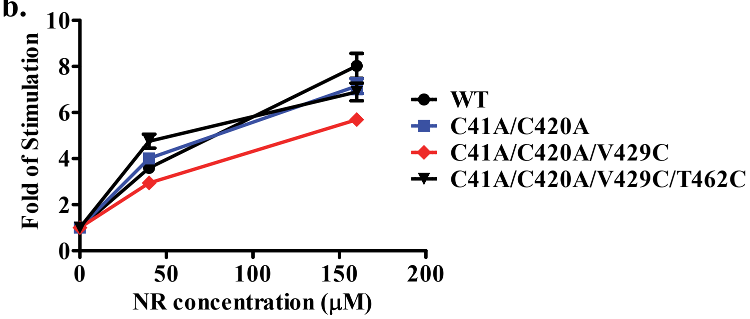

Supplementary Figure 7. The cysteine modifications used in the FRET and EPR analysis have little influence on BiP's biochemical activities.

a, The intrinsic ATPase and peptide-binding activities of the site-directed cysteine mutations in BiP. The single-turnover ATPase assay was carried out at 20°C, and the catalytic constants (k_{cat}) were calculated after fitting the data using Prism. Peptide substrate binding affinities were assayed using fluorescence polarization with the NR peptide, and the dissociation constants (K_d) were calculated by fitting one-site binding equation using Prism.

b, The allosteric coupling in BiP proteins was analyzed using the NR peptide stimulation on the ATPase activity. The intrinsic ATPase activities were set as 1. Fold of stimulation was plotted.

Supplementary Figure 8

BiP	BiP-ATP2		BiP-ATP		BiP-SBD		DnaK	DnaK-ATP		DnaK-SBD		Sse1	Sse1-ATP	
	φ(°)	ψ(°)	φ(°)	ψ(°)	φ(°)	ψ(°)		φ(°)	ψ(°)	φ(°)	ψ(°)		φ(°)	ψ(°)
T423	-63	130	-58	133	-66	132	S398	-68	136	-71	147	S401	-74	133
L424	-101	134	-100	148	-107	143	L399	-99	125	-121	136	V402	-123	124
G425	-136	164	-154	-176	-170	-166	G400	-142	-178	-168	-161	S403	-106	153
I426	-126	151	-128	155	-128	162	I401	-134	154	-134	163	Y404	-117	141
E427	-120	112	-77	137	-86	127	E402	-83	116	-83	129	S405	-124	162
T428	-92	120	-134	159	-108	177	T403	-108	166	-116	173	W406	-155	167
V429	-62	125	-26	130	-55	126	M404	-43	134	-50	124	D407	-62	130
G430	72	14	87	-137	104	11	G405	102	-4	110	-9			
G431	-134	-158	-140	66	64	43	G406	75	20	76	34			
V432	-83	153	-96	130	-99	124	V407	-86	154	-106	147	D414	-122	31
M433	-109	131	-77	88	-73	117	M408	-90	172	-89	131	H415	-170	137
T434	-154	146	-81	105	-112	117	T409	-109	118	-112	122	M416	-172	132
K435	-64	143	-58	146	-65	130	T410	-76	132	-83	130	E417	-74	134
L436	-120	-55	-132	-53	-111	-56	L411	-104	-41	-101	-41	V418	-119	-51
I437	-134	112	-128	113	-114	112	I412	-133	116	-121	118	F419	-117	100

Supplementary Figure 8. Backbone conformation of the β1-$L_{1,2}$-β2 segment in Hsp70 structures. The ψ and φ angles of Gly425 and Gly431 are highlighted in red.

Supplementary Figure 9

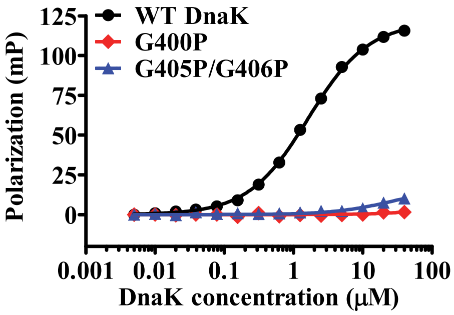

Supplementary Figure 9. The G400P and G405P/G406P mutations drastically reduced the peptide-binding activity of DnaK. The fluorescence polarization assay for peptide binding activity was carried out on the DnaK proteins in the same way as in Fig. 5**b**.

Supplementary Figure 10

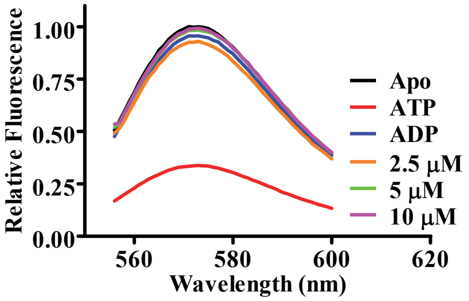

Supplementary Figure 10. ERdj3 has little effect on the fluorescence intensity of the TMR-labeled BiP in the presence of ADP. Assay was carried out essentially the same way as in Fig. 6**a** except that ADP was added in place of ATP.

Supplementary Figure 11

a.

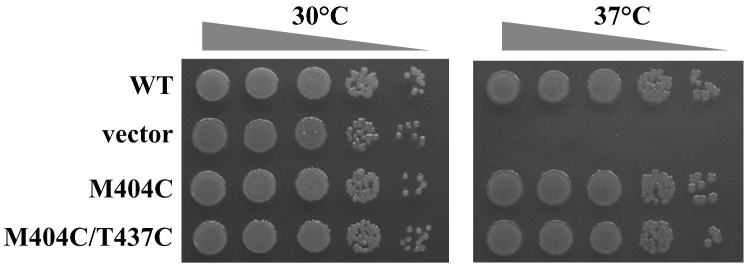

b.

DnaK proteins	k_{cat} (x10^{-3} min^{-1})	K_d (μM)
WT	27.88±2.18	1.70±0.07
C15A/M404C/T437C	28.03±2.95	2.12±0.07

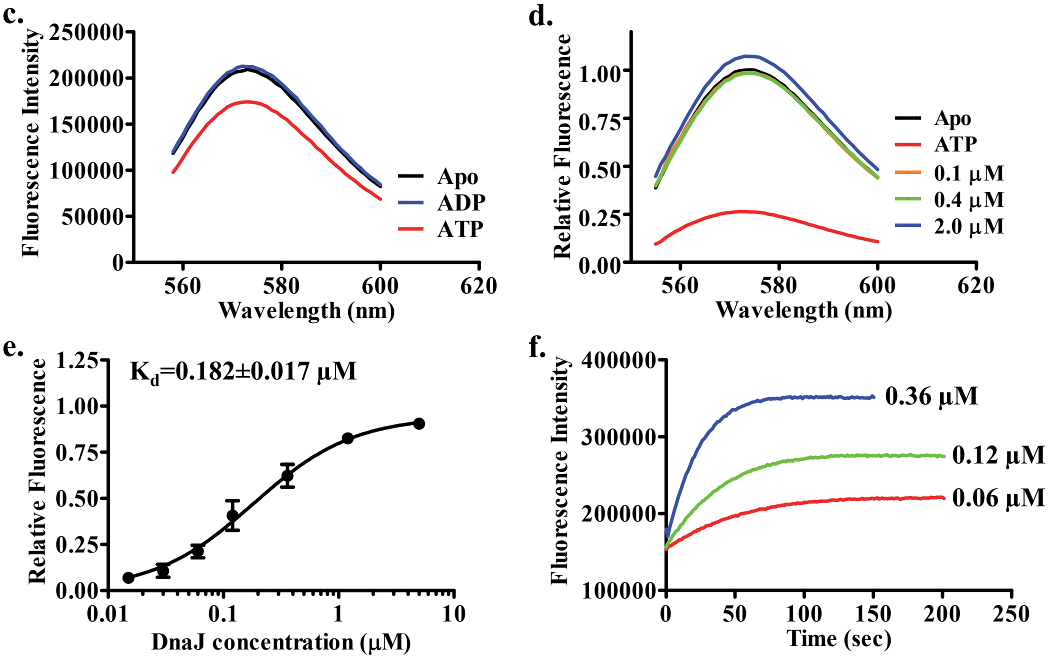

Supplementary Figure 11

Supplementary Figure 11. Conserved dynamics of $L_{1,2}$ in DnaK.

a, Neither M404C nor T437C modification has appreciable impact on the *in vivo* function of DnaK. Growth test of DnaK mutants in Hsp70-deficient *E.coli* was carried out. Serial dilutions of fresh cultures from fresh transformants were spotted onto plates and incubated at 37 °C with a 30 °C duplicate plate as growth control. WT DnaK and empty vector were used as positive and negative controls, respectively.

b, Both the intrinsic ATPase and peptide-binding activities of DnaK are largely unaffected by the M404C and T437C modifications. Assays were performed at room-temperature. For the peptide binding activity, fluorescence polarization assay with the model peptide NR was used and dissociation constants (K_d) were calculated. Single-turnover ATPase assay was used to determine the catalytic constants (k_{cat}).

c, The G400P mutation almost abolished the ATP-induced quenching of the TMR-labeled DnaK's fluorescence. Fluorescence emission spectra were collected in the same way as in Fig. 7**a**.

d, DnaJ has little influence on the fluorescence of the TMR-labeled DnaK in the absence of ATP. Assay was carried out essentially the same way as in Fig. 7**c** except that ATP was not included.

e, Dissociate constant (K_d) of DnaJ for DnaK in the FRET assay. Relative fluorescence was calculated by setting the fluorescence intensities of Apo and ATP alone at 573 nm (the peak emission) as 1 and 0, respectively.

f, The kinetics of DnaJ-induced opening of $L_{1,2}$. Assays were performed on DnaJ and the TMR-labeled DnaK in essentially the same way as in Fig. 6**c**. The concentrations of DnaJ were labeled on the right.
